# Role of gelatamp over absorbable gelatin sponge in impacted mandibular third molar surgery

**DOI:** 10.6026/973206300214534

**Published:** 2025-12-15

**Authors:** Udupikrishna Joshi, K Divya Vani, Satishkumar G Patil, Anand Mangalgi, Kavyashree Sagare, Ashwin Shah

**Affiliations:** 1 Department of Oral and Maxillofacial Surgery, H.K.E.S's S. Nijalingappa Dental College, Kalaburagi, Karnataka, India

**Keywords:** Gelatamp, gelatin sponge, third molar extraction, postoperative pain, hemostasis, oral surgery

## Abstract

Impacted mandibular 3rd molar extractions are linked with a modest incidence of postoperative difficulties. Several procedures such as pressure dressings, atraumatic surgical techniques and use of hemostatic material to minimize these complications have been employed. Therefore, it is of interest to assess the effectiveness of Gelatamp and conventional Gelfoam in reducing bleeding, postoperative pain and swelling after mandibular third molar surgery. Hence, a total of 21 patients undergoing bilateral mandibular third molar extractions were divided into, Group A Gelatamp and Group B Gelfoam. Postoperative outcomes such as bleeding, pain (VAS) and swelling were assessed after extraction of third molar. We observed that Gelatamp showed superior clinical efficacy over absorbable gelatin sponge in reducing postoperative pain, early bleeding andreduce swelling.

## Background:

Oral and Maxillofacial Surgeons frequently perform impacted mandibular third molar extractions, which are associated with a moderate incidence of postoperative complications, reported at approximately 10% [1]. Common sequelae include pain, bleeding, swelling and postoperative infection [2]. Although strict sterilization protocols have reduced infection rates, patient discomfort remains a significant concern [3]. To minimize these complications, several strategies have been employed, including pressure dressings, oral corticosteroids, atraumatic surgical techniques, irrigation solutions, proteolytic enzymes and specialized postoperative dressings [4, 5]. Despite their effectiveness, many of these methods carry drawbacks or adverse effects. Bleeding, in particular, remains a frequent intraoperative and postoperative challenge, arising from trauma to soft tissues, bone, or blood vessels. It may be exacerbated by systemic conditions (e.g., hypertension, anticoagulant therapy) or local factors such as inflammation [6]. While mechanical methods such as gauze pressure and suturing provide initial hemostasis, adjunctive use of local hemostatic agents is often necessary when bleeding persists or the risk of complications is high [7]. Gelatin sponge (Gelfoam) is a widely used hemostatic material prepared from purified porcine skin gelatin. With its porous, spongy texture and ability to absorb many times its weight in blood, it provides a physical scaffold that promotes platelet aggregation and clot formation. Absorbable type resorbs within 4-6 weeks and it is non-antigenic, supports natural healing while minimizing the risk of foreign body reaction [8].

Gelatamp, a silver-impregnated gelatin sponge (95% gelatin, 5% colloidal silver), combines the hemostatic properties of gelatin with broad-spectrum antimicrobial activity. The sustained release of silver ions provides prolonged antibacterial protection against oral pathogens, including antibiotic-resistant strains. This dual action not only stabilizes the clot but also minimizes the risk of infection, dry socket and secondary bleeding [9]. In the context of mandibular third molar extractions, gelatin sponge and Gelatamp provide several clinical advantages beyond hemostasis. They contribute to reduced postoperative pain, improved bleeding control and decreased swelling. By offering a stable, antimicrobial and absorbable matrix, these agents significantly improve patient comfort and overall surgical outcomes [10]. Therefore, it is of interest to assess the effectiveness of Gelatamp and conventional Gelfoam in reducing bleeding, postoperative pain and swelling after mandibular third molar surgery.

## Materials and Methodology:

The present study was conducted in the department of Oral and Maxillofacial Surgery at HKES's S. Nijalingappa Institute of Dental Sciences and Research, Gulbarga, Karnataka, after obtaining the ethical clearance from the institute and informed consent from all the participants. The participants were selected from the Outpatient Department of Oral and Maxillofacial Surgery. The sample size estimation was carried out using Master 2.0 software developed by the Department of Biostatistics, Christian Medical College and Vellore. Based on statistical parameters and desired precision, a minimum sample size of 21 subjects was determined to be adequate to achieve a 95% confidence level, ensuring sufficient statistical power and reliability of the study results. The participants were selected according to specific inclusion and exclusion criteria. The inclusion criteria comprised patients aged above 18 years to 32 years, those presenting with impacted mandibular third molars and individuals willing to voluntarily participate in the study after providing informed consent. The exclusion criteria included patients with odontogenic cysts or other pathological lesions associated with the impacted tooth and those unwilling to participate in the study.

## Postoperative assessment parameters:

## Postoperative Bleeding:

Hemostasis was evaluated using the Maani *et al.* Bleeding Index: Grade 0: No bleeding, Grade 1: Slight oozing, stops spontaneously or with pressure, Grade 2: Clinically significant bleeding, Grade 3: Bleeding after clot formation and Grade 4: Excessive bleeding not controlled by local measures.

## Pain:

Pain intensity was assessed using a 10-point Visual Analogue Scale (VAS), where0 = no pain, 5 = moderate pain and 10 = worst pain.

## Swelling:

Swelling will be quantified with a five-line linear measurement using the plastic scale as follows: (Line 1) from the tragus of the ear to the point on the commissure of the lip, (Line 2) from the tragus of the ear to the pogonion, (Line 3) from the tragus of the ear to the lateral canthus of the eye. (Line 4) from the lateral canthus of the eye to the most Inferior point on the angle of the mandible and (Line 5) the most inferior point on the mandible's angle to the nasal bone's midpoint.

## Procedure:

Patients who met the inclusion criteria were enrolled in the study and alienated into two groups: Group A (Gelatamp) and Group B (absorbable gelatin sponge). Each patient was thoroughly informed about the surgical procedure and only those who gave written consent were included. A detailed case record was maintained for every participant. All surgeries were performed under local anesthesia by the same experienced oral and maxillofacial surgeon, following standardized surgical protocols. The procedure involved making an incision, reflecting a mucoperiosteal flap, removing bone using 703 burs under saline irrigation, extracting the tooth with elevators and forceps, followed by curettage, smoothing of bony edges and placement of either Gelatamp or a gelatin sponge in the socket before closing the wound. Patients were given clear postoperative instructions and prescribed a five-day course of antibiotics, analgesics and anti-inflammatory medication. Follow-up evaluations were carried out on the 2nd, 5th and 7th postoperative days. Postoperative outcomes after extraction of third molar were assessed such as; bleeding, pain (VAS) and swelling. Gelatamp demonstrated superior clinical efficacy over absorbable gelatin sponge in reducing postoperative pain, early bleeding and reduce swelling. The obtained data was statistically evaluated using SPSS statistical software version 24.0 at p<0.05.

## Results:

The clinical study included 21 patients, having bilateral mandibular third molar impaction sites indicated for surgical removal. Thus total of 42 sites were included. These were evenly divided into two groups: Group A (Gelatamp) and Group B (absorbable gelatin sponge/Gelfoam), with 21 sites in each. The mean age of the participants was 24.23 ± 2.72 years and ranging from 18 to 32 years, with the majority (76.2%) falling in the 18-25 age groups. The study showed a female predominance, with 71.4% females and 28.6% males, yielding a male-to-female ratio of 1:2.5. Tooth sites (38 and 48) were nearly equally distributed between both groups, with no statistically significant difference (P = 0.354). Bleeding was assessed at multiple intervals postoperatively. While no significant difference was found between the groups at 0, 15 and 30 minutes, Gelatamp showed significantly better hemostatic control at the 5-minute mark (P = 0.0176), suggesting its superior early hemostatic effect (Table 1 - see PDF). Pain was consistently lower in the Gelatamp group across all time points. On the 2nd postoperative day, the mean pain score in the Gelatamp group was significantly lower (5.81 ± 1.66) than in the Gelfoam group (7.71 ± 2.00) with P = 0.002. By day 5, pain scores were 3.81 ± 1.91 and 4.90 ± 1.37, respectively (P = 0.039) and on day 7, the difference widened further with scores of 0.76 ± 1.09 in the Gelatamp group and 2.38 ± 1.53 in the Gelfoam group (P < 0.001), indicating a highly significant reduction in pain (Figure 1 - see PDF). Swelling was evaluated at five facial reference lines (L1-L5) on days 2, 5 and 7. On the 2nd day, swelling along the L1 line was significantly lower in the Gelatamp group (P = 0.020), but no significant variations were found at other lines or time points (Figure 2 - see PDF).

## Discussion:

The surgical removal of impacted mandibular third molars is among the most frequently performed procedures in oral and maxillofacial surgery and is often associated with postoperative complications, reported in approximately 10% of cases [1]. Pain, infection, swelling and trismus are the most common sequelae. While advances in sterilization protocols and surgical techniques have reduced infection rates, patient morbidity following third molar surgery remains a significant concern. Therefore, the choice of local adjuncts for hemostasis and wound protection is critical for optimizing outcomes [2, 3]. The present study comparatively evaluated Gelatamp, a sterile, resorbable gelatin sponge impregnated with colloidal silver and Gelfoam, a conventional absorbable gelatin sponge, in terms of their effectiveness in managing postoperative complications. The findings demonstrated that Gelatamp conferred significant clinical benefits, particularly in early hemostasis, pain reduction and enhanced wound healing. Hemostasis is fundamental in oral surgical procedures where vascularity and salivary contamination may destabilize clot formation. Both Gelfoam and Gelatamp facilitated hemostasis [11]; however, Gelatamp demonstrated significantly superior results at the critical 5-minute interval postoperatively (P = 0.0176). This effect is attributed to the dual mechanism of action: (i) the gelatin matrix providing mechanical tamponade and a scaffold for platelet aggregation and (ii) colloidal silver reducing microbial interference and fibrinolysis [9]. Maani *et al.* (2015) reported that Gelatamp effectively controlled bleeding in patients on anticoagulant therapy without the need to discontinue medication [8]. These findings align with the present study, confirming Gelatamp's enhanced hemostatic potential. Postoperative pain substantially influences recovery and patient satisfaction. In this study, patients treated with Gelatamp consistently reported lower pain scores on postoperative days 2, 5 and 7, with statistical significance by day 7 (P < 0.001). It has been reported that, antibacterial activity of colloidal silver, limiting the microbial colonization and subsequent inflammation Moist wound environment created by the gelatin matrix, supports nerve protection and tissue regeneration [12]. Physical shielding of the extraction socket, reduce mechanical irritation from food debris or tongue movement [13]. Rahuman concluded that the use of gelatin sponge in the extraction sockets of impacted third molars, in the absence of excessive bleeding, reduces postoperative pain when compared to a non-packed control site [14]. Sing *et al.* are not in agreement with the assertion that haemostatic agents can serve as substitutes for bone grafts [15]. Postoperative edema is an expected response but contributes significantly to patient discomfort. In the present study, Gelatamp significantly reduced swelling on the 2nd postoperative day at the L1 anatomical line (P = 0.020), though results across other measurements were inconsistent. This suggests localized modulation of the inflammatory response, likely influenced by anatomical factors.

## Conclusion:

Gelatamp, a colloidal silver-impregnated gelatin sponge, offers superior clinical benefits over conventional absorbable gelatin sponge (Gelfoam) in the postoperative management of impacted mandibular third molar extractions. Gelatamp achieved significantly faster hemostasis at the critical 5-minute interval, reduced postoperative pain throughout the first week. It also contributed to reduce early swelling at selected anatomical points.

## Funding:

Nil
